# PReS-FINAL-2076: Single centre experience of biologics in clinical practice of systemic juvenile idiopatic arthritis

**DOI:** 10.1186/1546-0096-11-S2-P88

**Published:** 2013-12-05

**Authors:** MI Kaleda, IP Nikishina, SR Rodionovskaya, AN Shapovalenko

**Affiliations:** 1 Pediatrics, "Scientific Research Institute of Rheumatology named after V.A.Nasonovoy" RAMS, Moscow, Russian Federation

## Introduction

A distinctive feature of systemic juvenile idiopathic arthritis (sJIA) is its high resistance to therapy of DMARDs and biologics (B) used as the 1^st ^line before the advent of IL-1 and IL-6 inhibitors.

## Objectives

Analysis of experience of using B by patients (Pts) with sJIA in a single centre during the last 10 years.

## Methods

Our retrospective study included 59 Pts (20 boys, 39 girls), who used B. All Pts had diagnosis of sJIA, established according to the ILAR classification criteria. The clinical characteristic at the time of 1^st ^prescription: mean age of 6.25, range 2.0-17.75 yrs; mean disease duration of 4.5, range 0.3-15.9 yrs; 61% of Pts had active systemic features, 98.3% - arthritis. The previous therapy included NSAIDs (95%), steroids (76%), DMARDs (methotrexate (MTX) alone - 18.6%, 2 DMARDs - 68%, 3 DMARDs consecutive - 12%). As the 1^st ^B used: infliximab (INF) - 32%, tocilizumab (TCZ) - 39%, etanercept (ETA) - 10%, adalimumab (ADA) - 9%, abatacept (ABA) - 5%, rituximab (RTM) - 5%. 39 Pts received only 1 B: by 12.8% - INF or ADA, 53.8%TCZ, 15.4%ETA, by 2.6% RTM or ABA. 22 Pts received 2 B successively, the 2^nd ^B used: 50%TCZ, 13%ETA, 14%ADA, 14%ABA, 9%RTM. 9 Pts received 3 B, the 3d B used: 45%TCZ, 22%ABA, 22% canacinumab (CAN), 11%ADA. 2 Pts received successively 4 B (INF-ADA-TCZ-ABA, INF-TCZ-ABA-ETA). The reasons for substitution therapy were serious adverse effects (SAE), subsequent loss of effect. We did not use anakinra because it is not available in Russia.

## Results

43 Pts continue treatment: 69.7%TCZ, 9.2%ETA, 6.9%INF, by 4.6%ADA, ABA and CAN. The response of therapy is more than 30-50% by the ACRpedi criteria. Stable improvement allowed to decrease prednisolone (PR) dose in all Pts, to cancel PR in 9.3% Pts, to cancel NSAIDs in 14% Pts. 11 Pts have status of inactive desease (8-TCZ, 2-INF, 1-ETA). Adverse events and laboratory abnormalities included neutropenia (was observed in 6 Pts receiving TCZ during 1-3 days after infusion) and elevated transaminases (1-ETA, 4-TCZ). 6 Pts increased the weight (4-TCZ, 2-INF). We have observed some SAE: infusion reaction (3-TCZ, 2-INF); severe infections - sepsis (2 Pts-RTM), varicella (1-INF, 2-TCZ, 1-ABA), atypical pneumonia (1-TCZ, 1-ETA), tuberculosis (1- ETA, 1-TCZ). Pts had worse results of treatment in a long duration of the disease, with the damage of hip and the cervical spine, in the presence of synovial cysts.

## Conclusion

TCZ is the best choice among B at sJIA in Pts with ongoing systemic manifestations. The use of TNF inhibitors in sJIA is characterized by high efficiency in the initial stages of therapy and subsequent loss of effect with a high frequency of systemic exacerbations in most Pts. Careful monitoring provided an acceptable safety profile of TCZ, TNF-inhibitors and ABA in the Pts with sJIA.

## Disclosure of interest

None declared.

